# Cannabis addiction in Tunisia: sociodemographic profile and neuropsychological complications

**DOI:** 10.1192/j.eurpsy.2024.245

**Published:** 2024-08-27

**Authors:** E. Smaoui, D. Mnif, R. Ouali, R. Sellami, I. Feki, I. Baati, J. Masmoudi

**Affiliations:** Psychiatry, CHU Hédi Chaker, Sfax, Tunisia

## Abstract

**Introduction:**

Cannabis consumption constitutes a public health problem both because of its serious repercussions and complications and the psychological and social problems it causes.

**Objectives:**

Our objective was to assess the level of cannabis dependence in consumers receiving care at the Sfax detoxification center in Tunisia, to describe the sociodemographic profile of these consumers and the neuropsychological complications that may be caused.

**Methods:**

We conducted a cross-sectional study, over a period of 13 months (September 2020 to October 2021), among cannabis users consulting the Sfax detoxification center in Tunisia. We used the Cannabis Abuse Screening Test (CAST) in order to detect a “problematic” cannabis use, along with a clinical information sheet to collect epidemiological and clinical data. All patients gave their free and informed oral consent to participate in the survey while ensuring anonymity.

**Results:**

We included 38 patients. The average age was 26 years old with a median age of starting cannabis use at 17 years old. The sex ratio was 8.5 with an over-representation of men. Most of the subjects were single, lived with their family and had a secondary school education. Consumption was daily for the majority of patients (68.5%) with an average quantity of 4 joints/day. According to the CAST scale, 36 users (94.7%) had problematic cannabis use. The factors favouring cannabis consumption were stress and anxiety in 34 patients (89.5%) followed by depression and the festive atmosphere in 14 subjects (36.8%) each. Among the participants, 26.3% had a psychiatric history including depression (5.3%), a psychopathic personality disorder (10.5%) and cannabis-induced psychotic disorder (10.5%). History of psychiatric hospitalization and history of suicide attempt were found in 21.1% and 26.3% of the patients respectively. Concerning the complications caused by cannabis, 68.4% of the patients described a phenomenon of tolerance, while 63.2% reported the sensation of craving. Psychotic symptoms such as delirium and/or hallucinations were found in 6 patients (15.8%) and 8 subjects (21.1%) reported a history of overdose in the form of cannabis psychosis. Chronic complications were an amotivational syndrome (63.2%) and social disintegration (52.6%). Treatment of cannabis dependence was considered effective with total withdrawal in 31.6% of subjects. Weaning was partial in 42.1% of the patients.

**Conclusions:**

Cannabis use is emerging as one among many interacting factors that can affect psychological and physical health, with an impact on various levels including mood, neurocognition and general health. Although studies have shown functional brain mechanisms underlying the effects of cannabis, the exact mechanisms remain unclear. Overall, treatment for substance use disorders generally prevents these complications and improves prognosis.

**Disclosure of Interest:**

None Declared

